# Supplementary material for: Unravelling Heavy Metal Dynamics in Soil and Honey: A Case Study from Maramureș Region, Romania
Source: Foods. 2023 Sep 26;12(19):3577. doi: 10.3390/foods12193577 (PMC10573013; doi:10.3390/foods12193577)
Supplement: Supplementary file 1 [file foods-12-03577-s001.zip › foods-2591214-supplementary.pdf]

Table S1. Information about soil sampling sites from Maramureș, Romania

Sample code	Number of samples	Harvest period	Soil details	Sampling depth (cm)	Geographical origin	Distance from the source of pollution (~) km	Area/ Country	Year of harvest	Environment	Anthropogenic influence
Soil samples exposed to anthropogenic sources of heavy metals pollution										
$S_{1\text{-}2019}$	3	June July	The collected sample corresponds to the hive where chestnut honey was obtained	0 – 10 cm	Tăuții de Sus	3.5 km to the settling ponds *Aurul*	Maramureș Country/ Romania	2019	Semi-rural	Near (~ 3.5 km distance) to the settling pond mining (decant pond) *Aurul*[1] from Tăuții de Sus
$S_{1\text{-}2020}$	2	June July	The collected sample corresponds to the hive where chestnut honey was obtained	0 – 10 cm	Tăuții de Sus	4.0 km to the settling ponds *Aurul*	Maramureș Country/ Romania	2020	Semi-rural	Near (~ 4.0 km distance) to the settling pond mining (decant pond) *Aurul*[1] from Tăuții de Sus
$S_{1\text{-}2021}$	2	June July	The collected sample corresponds to the hive where chestnut honey was obtained	0 – 10 cm	Tăuții de Sus	3.8 km to the settling ponds *Aurul*	Maramureș Country/ Romania	2021	Semi-rural	Near (~ 3.8 km distance) to the settling pond mining (decant pond) *Aurul*[1] from Tăuții de Sus
$S_{2\text{-}2018}$	1	June July	The collected sample corresponds to the hive where chestnut honey was obtained	0 – 10 cm	Tăuții Măgherăuș	9.0 km to the Nistru mine and 6.0 km to the Băița mine	Maramureș Country/ Romania	2018	Rural	Near (~ 9.0 km distance) to the Nistru mine and (~ 6.0 km distance) to Băița mine
$S_{2\text{-}2019}$	1	June July	The collected sample corresponds to the hive where chestnut honey was obtained	0 – 10 cm	Tăuții Măgherăuș	11.0 km to the Nistru mine and 8.0 km to	Maramureș Country/ Romania	2019	Rural	Near (~ 11.0 km distance) to the Nistru mine and (~ 8.0 km distance) to Băița mine

						the Băița mine				
S_{2-2021}	2					11.2 km to the Nistru mine and 8.3 km to the Băița mine		2021	Rural	Near (~ 11.2 km distance) to the Nistru mine and (~ 8.3 km distance) to Băița mine
S_{3-2019}	2	June July	The collected sample corresponds to the hive where chestnut honey was obtained	0 – 10 cm	Baia Mare	8.0 km to the settling ponds *Aurul*	Maramureș Country/Romania	2019	Semi-rural	Near (~ 8.0 km distance) to the settling pond mining (decant pond) *Aurul*[1] from Tăuții de Sus
S_{3-2020}	2							2020		
S_{3-2021}	3							2021		
S_{4-2021}	1	June July	The collected sample corresponds to the hive where polyfloral honey was obtained	0 – 10 cm	Tăuții Măgherăuș	6.5 km to the settling ponds *Aurul*		2021	Semi-rural	Near (~ 8.0 km distance) to the settling pond mining (decant pond) *Aurul*[1] from Bozânta Mare

S_{5-2019}	2	July August	The collected sample corresponds to the hive where polyfloral honey was obtained	0 – 10 cm	Baia Sprie	8.0 km to the Herja mine	Maramureș Country/ Romania	2019	Rural	Near (~ 8.0 km distance) to the Herja mine
S_{6-2020}	1	July August	The collected sample corresponds to the hive where acacia honey was obtained	0 – 10 cm	Baia Mare	8.0 km to the settling ponds *Aurul*	Maramureș Country/ Romania	2020	Semi-rural	Near (~ 8.0 km distance) to the settling pond mining (decant pond) *Aurul*[1] from Tăuții de Sus
S_{6-2021}	1	July August						2021	Semi-rural	
S_{7-2017}	1	July August	The collected sample corresponds to the hive where polyfloral honey was obtained	0 – 10 cm	Baia Borșa	5.1 km to the Băile Borșa mining flotation	Maramureș Country/ Romania	2017	Rural	Near (~ 5.1 km distance) to the Băile Borșa mining flotation
S_{7-2019}	1	July August		0 – 10 cm	Baia Borșa	7.3 km to the Băile Borșa mining flotation		2019	Rural	Near (~ 7.3 km distance) to the Băile Borșa mining flotation
S_{7-2020}	1	July August	The collected sample corresponds to the hive where polyfloral	0 – 10 cm	Baia Borșa	9.0 km to the Băile Borșa mining flotation		2020	Rural	Near (~ 9.0 km distance) to the Băile Borșa mining flotation

			honey was obtained							
$S_{7\text{-}2021}$	1	July August	The collected sample corresponds to the hive where polyfloral honey was obtained	0 – 10 cm	Baia Borșa	8.0 km to the Băile Borșa mining flotation	Maramureș Country/ Romania	2021	Rural	Near (~ 8.0 km distance) to the Băile Borșa mining flotation
$S_{8\text{-}2021}$	2	July August	The collected sample corresponds to the hive where polyfloral honey was obtained	0 – 10 cm	Satulung	5.0 km to the European Road (E 58)	Maramureș Country/ Romania	2021	Semi-rural	Near (~ 5.0 km distance) to the European Road (E 58)
$S_{9\text{-}2020}$	1	July August	The collected sample corresponds to the hive where polyfloral honey was obtained	0 – 10 cm	Săcălășeni	8.0 km to the European Road (E 58)	Maramureș Country/ Romania	2020	Semi-rural	Near (~ 8.0 km distance) to the European Road (E 58)
					Background soil samples					
$S_{10\text{-}2020}$	2	August	The collected sample corresponds to the hive	0 – 10 cm	Groșii Țibleșului	-		2020	Rural	-

			where polyfloral honey was obtained							
$S_{11\text{-}2018}$	2	August	The collected sample corresponds to the hive where polyfloral honey was obtained	0 – 10 cm	Vișeu de Sus	-	Maramureș Country/ Romania	2018	Rural	-
$S_{11\text{-}2019}$	1	August	The collected sample corresponds to the hive where polyfloral honey was obtained	0 – 10 cm	Vișeu de Sus	-	Maramureș Country/ Romania	2019	Semi-rural	-
$S_{11\text{-}2020}$	2	August	The collected sample corresponds to the hive where polyfloral honey was obtained	0 – 10 cm	Vișeu de Sus	-	Maramureș Country/ Romania	2020	Urban	-

$S_{11-2021}$	1	August	The collected sample corresponds to the hive where polyfloral honey was obtained	0 – 10 cm	Vișeu de Sus	-	Maramureș Country/ Romania	2021	Non-urban area		-

S = soil sample (it does not refer to sites); the collection of soil samples from 9 sites was carried out strictly according to the type of honey and to the year of production, e.g., from Tăuții de Sus site, three main soil samples were collected (subsamples n = seven), these correspond to the chestnut honey obtaining in the 2019-2021 years (e.g., for the chestnut honey produced in 2019 were collected three soil subsamples); N° of samples = number of soil samples harvested form each site, depending of the type of honey and the year in which it was obtained.

Table S2. Information about honey sampling sites from Maramureș, Romania

Sample code	Number of samples	Harvest period	Honey details	Denomination	Geographical origin	Distance from the source of pollution (~) km of the hives	Area/ Country	Year of harvest	Type of extraction	Environment	Anthropogenic influence	Bee species
Honey samples exposed to anthropogenic sources of heavy metals pollution												
H_{1-2019}	2	June July	Raw artisan honey	Chestnut honey	Tăuții de Sus	3.5 km to the settling ponds *Aurul*	Maramureș Country/Romania	2019	Manual	Semi-rural	Near (~ 3.5 km distance) to the settling pond mining (decant pond) *Aurul*[1] from Tăuții de Sus	*Apis mellifera*
H_{1-2020}	1							2020				
H_{1-2021}	3							2021				
H_{2-2018}	1	June July	Raw artisan honey	Chestnut honey	Tăuții Măgherăuș	9.0 km to the Nistru mine and		2018	Manual	Rural	Near (~ 9.0 km distance) to the	*Apis mellifera*
H_{2-2019}	1							2019				

H_{2-2021}	1					6.0 km to the Băița mine	2021			Nistru mine and (~6.0 km distance) to Băița mine	
H_{3-2019}	2	June July	Raw artisan honey	Chestnut honey	Baia Mare	8.0 km to the settling ponds *Aurul*	2019	Manual	Semi-rural	Near (~ 8.0 km distance) to the settling pond mining (decant pond) *Aurul*[1] from Tăuții de Sus	*Apis mellifera*
H_{3-2020}	2						2020				
H_{4-2021}	3	June July	Artisan honey	Polyfloral honey	Tăuții Măgherăuș	6.5 km to the settling ponds *Aurul*	2021	Mechanical	Semi-rural	Near (~ 6.5 km distance) to the settling pond mining (decant pond) *Aurul*[1] from Bozânta Mare	*Apis mellifera*
H_{5-2019}	1	July August	Artisan honey	Polyfloral honey	Baia Sprie	8.0 km to the Herja mine	2019	Manual	Rural	Near (~ 8.0 km distance) to the Herja mine	*Apis mellifera*
H_{6-2020}	2	July August	Artisan honey	Acacia	Baia Mare	8.0 km to the settling ponds *Aurul*	2020	Mechanical	Semi-rural	Near (~ 8.0 km distance) to the settling pond mining (decant pond) *Aurul*[1] from Tăuții de Sus	*Apis mellifera*
H_{6-2021}	1						2021				
H_{7-2017}	1	July August	Artisan honey	Polyfloral honey	Baia Borșa	5.1 km to the Băile Borșa	2017	Manual	Rural	Near (~ 5.1 km distance) to the Băile Borșa	*Apis mellifera*

H$_{7-2019}$	1					Mining Flatation		2019			Mining Flatation	
H$_{7-2020}$	1							2020				
H$_{7-2021}$	1							2021				
H$_{8-2021}$	2	July August	Artisan honey	Polyfloral honey	Satulung	5.0 km to the European Road (E 58)	Maramureș Country/Romania	2021	Mechanical	Semi-rural	Near (~ 5.0 km distance) European Road (E 58), with intense traffic of vehicles	*Apis mellifera*
H$_{9-2020}$	1	July August	Artisan honey	Polyfloral honey	Săcălășeni	8.0 km to the European Road (E 58)		2020	Mechanical	Semi-rural	Near (~ 8.0 km distance) European Road (E 58), with intense traffic of vehicles	*Apis mellifera*
Background honey samples												
H$_{10-2020}$	2	August	Artisan honey	Polyfloral honey	Groșii Țibleșului	-	Maramureș Country/Romania	2020	Mechanical	Rural	-	*Apis mellifera*
H$_{11-2018}$	3	August	Artisan honey	Polyfloral honey	Vișeu de Sus			2018	Manual	Rural	-	*Apis mellifera*

$H_{11\text{-}2019}$	1		2019	Mechanical	Semi-rural
$H_{11\text{-}2020}$	2		2020		Urban
$H_{11\text{-}2021}$	3		2021	Manual	Non-urban area

[1] Settling Pond Mining *Aurul* resulting from the processing of blank ores in the Baia Sprie Mine Preparation Plant and which contains approximately 13.5 tons of mine tailings stored in the 1965-2003 period. At present Settling Pond Mining *Aurul* is the property of the National Company Remin SA Baia Mare (*Compania Națională Remin SA Baia Mare*) [47].

Table S3. The program of the microwave oven Milestone START D Microwave Digestion System

Step	Target Temp (°C)	Pressure Max. (psi)	Temperature Ramp (min.)	Hold Time (min.)	Power (%)
			Soil		
1.	220	800	10	20	100
2.	220	800	10	15	60
3.	35-40	800	-	45 min. cooling	-
			Honey		
1.	200	800	10	20	100
2.	200	800	10	15	55
3.	35-40	800	-	45 min. cooling	-

Table S4. Instrumental (a) and data acquisition (b) parameters of ICP-MS

(a) Instrumental parameters		(b) Data acquisition parameters for quantitative mode	
RF power/W	1.4 kW	Measuring mode	Standard (Ar 5.0)<br>Q Cell (Collision Cell)<br>(He 6.0)
Argon (Ar) gas flow, Helium (He) gas flow		Point per peak	3
Nebulizer	1.0 L/min.	Scans/Replicate	7
Plasma gas low rate (Ar 5.0)	18.0 L/min.	Replicate/Sample	7
Auxiliary gas flow rate (He 6.0)	0.20 L/min.		
Lens voltage	37 V	Dwell time (ms)	3
Mirror lens right	32 V		
Mirror lens bottom	31 V		
Sample uptake rate	90 s	Integration time	1-5 ms
Temperature spray chamber		2.10 °C	
Background correction		2 points/peak	
Injector tube		quartz 2-mm id	
Sample cone		Sample Cone 4450	
Skimmer cone		Ni – Skimmer iCAP Q 0.5 mm insert version	
Nebulizer		MicroMist Nebulizer 0.4 mL/min.	

Table S5. Instrumental conditions for the determination of each element using the ICP-MS technique

Element	Correlation coefficient	LoD (μg/L)	LoQ (μg/L)	BEC (μg/L)	Element	Correlation coefficient	LoD (μg/L)	LoQ (μg/L)	BEC (μg/L)
Cu	0.9996	0.0414	1.545	1.258	Zn	0.9999	0.079	1.203	1.310
Pb	0.9996	0.231	0.231	0.649	Cd	0.9997	0.007	0.069	0.0031
Ni	0.9997	0.045	0.181	0.096	Co	0.9997	0.051	0.136	0.152
As	0.9999	0.006	0.743	0.018	Cr	0.9999	1.607	5.533	0.637
Hg	0.9999	0.043	0.1379	0.128					

LoD = Detection limit; LoQ = Quantification limit; BEC = Background equivalent concentration.

Table S6. Validation parameters of the analytical procedure for the determination of heavy metals (soil)

Element	Certified reference material analysis		Validation parameters	
	The result declared by de manufacture	The results obtained in our research	Recovery (%)	Uncertainty (%)
Cu (mg/kg)[a]	34.6 ± 0.7	32.45 ± 2.78	96.7	19
Zn (mg/kg)[a]	106 ± 3	100.1 ± 3.04	95.2	18
Pb (mg/kg)[b]	18.9 ± 0.5	19.16 ± 0.84	96.0	11
Cd (mg/kg)[a]	0.38 ± 0.01	0.37 ± 0.01	99.1	12
Ni (mg/kg)[a]	88 ± 5	85.8 ± 2.36	85.3	16
Co (mg/kg)[a]	13.4 ± 0.7	13.97 ± 0.75	95.5	21
As (mg/kg)[a]	17.7 ± 0.8	16.7 ± 0.94	92.6	22
Cr (mg/kg)[a]	103 ± 4	101.21 ± 2.36	98.8	18
Hg (mg/kg)[a]	1.4 ± 0.08	1.29 ± 0.02	98.7	13

1. [a] SRM – 2709a Standard Reference Material „San Joaquin Soil" Baseline Trace Element Concentration.

Table S7. Validation parameters of the analytical procedure for the determination of heavy metals (honey)

Element	Certified reference material analysis		Validation parameters	
	The result declared by de manufacture	The results obtained in our research	Recovery (%)	Uncertainty (%)
Cu (mg/kg)[a]	4.70 ± 0.14	4.42 ± 0.31	96.87	23
Zn (mg/kg)[a]	30.94 ± 0.55	31.84 ± 0.86	100.12	17
Pb (mg/kg)[b]	0.167 ± 0.015	0.164 ± 0.003	96.78	22
Cd (mg/kg)[a]	1.517 ± 0.027	1.687 ± 0.147	90.32	23
Ni (mg/kg)[a]	1.582 ± 0.041	1.784 ± 0.084	98.70	18
Co (mg/kg)[a]	0.5773 ± 0.071	0.6541 ± 0.321	107.18	24
As (mg/kg)[a]	0.1126 ± 0.024	0.1126 ± 0.062	117.89	16
Cr (mg/kg)[a]	1.988 ± 0.034	2.124 ± 0.070	92.69	21
Hg (mg/kg)[a]	0.0341 ± 0.0015	0.023 ± 0.017	95.84	10

2. [a] NIST – 1573a Tomato Leaves Standard Reference Materials; [b] NIST – 1575a Pine Needles (*Pinus taeda*) Standard Reference Materials

Table S8. The influence of factors on the concentration of heavy metals in the soil sites from Maramureș, Romania

Element / Factor	Cu	Zn	Pb	Cd	Ni	Co	As	Cr	Hg
		Sig.							
Area	***	***	***	***	***	***	***	***	-
Year	ns	ns	ns	ns	ns	ns	ns	ns	-
Distance from the sources of pollution	***	***	***	***	***	***	***	***	-
Anthropogenic influence	***	***	***	***	***	***	***	***	-
Environment	*	ns	ns	ns	ns	ns	ns	ns	-
Soil details*	***	***	***	***	***	***	***	***	-

* The collected soil sample corresponds to the type of analyzed honey (chestnut, polyfloral, and acacia honey).

Table S9. The influence of factors on the concentration of heavy metals in honey sites from Maramureș, Romania

Element / Factor	Cu	Zn	Pb	Cd	Ni	Co	As	Cr	Hg
			Sig.						
Area	***	***	***	***	***	-	-	***	-
Year	ns	ns	ns	ns	ns	-	-	ns	-
Distance from the sources of pollution	***	***	***	***	***	-	-	***	-
Anthropogenic influence	***	***	***	***	***	-	-	***	-
Environment	**	ns	ns	ns	ns	-	-	ns	-
Honey details*	***	***	***	***	***	-	-	***	-

* The collected soil sample corresponds to the type of analyzed honey (chestnut, polyfloral, and acacia honey).
